# Editorial: Peri-operative care in cardiac surgery

**DOI:** 10.3389/fcvm.2024.1524314

**Published:** 2024-11-26

**Authors:** Marco Pocar, Cristina Barbero, Andrea Costamagna, Mauro Rinaldi, Luca Brazzi

**Affiliations:** ^1^ Cardiovascular and Thoracic Department, ‘Città della Salute e della Scienza’ University Hospital, Turin, Italy; ^2^ Department of Surgical Sciences, University of Turin, Turin, Italy; ^3^ Department of Anesthesia, Intensive Care and Emergency, ‘Città della Salute e della Scienza’ University Hospital, Turin, Italy

**Keywords:** perioperative care, cardiac surgery, outcome, quality of care, risk - benefit, complications, minimally invasive approach, new technology (NT)

**Editorial on the Research Topic**
Peri-operative care in cardiac surgery

**Figure 1:**
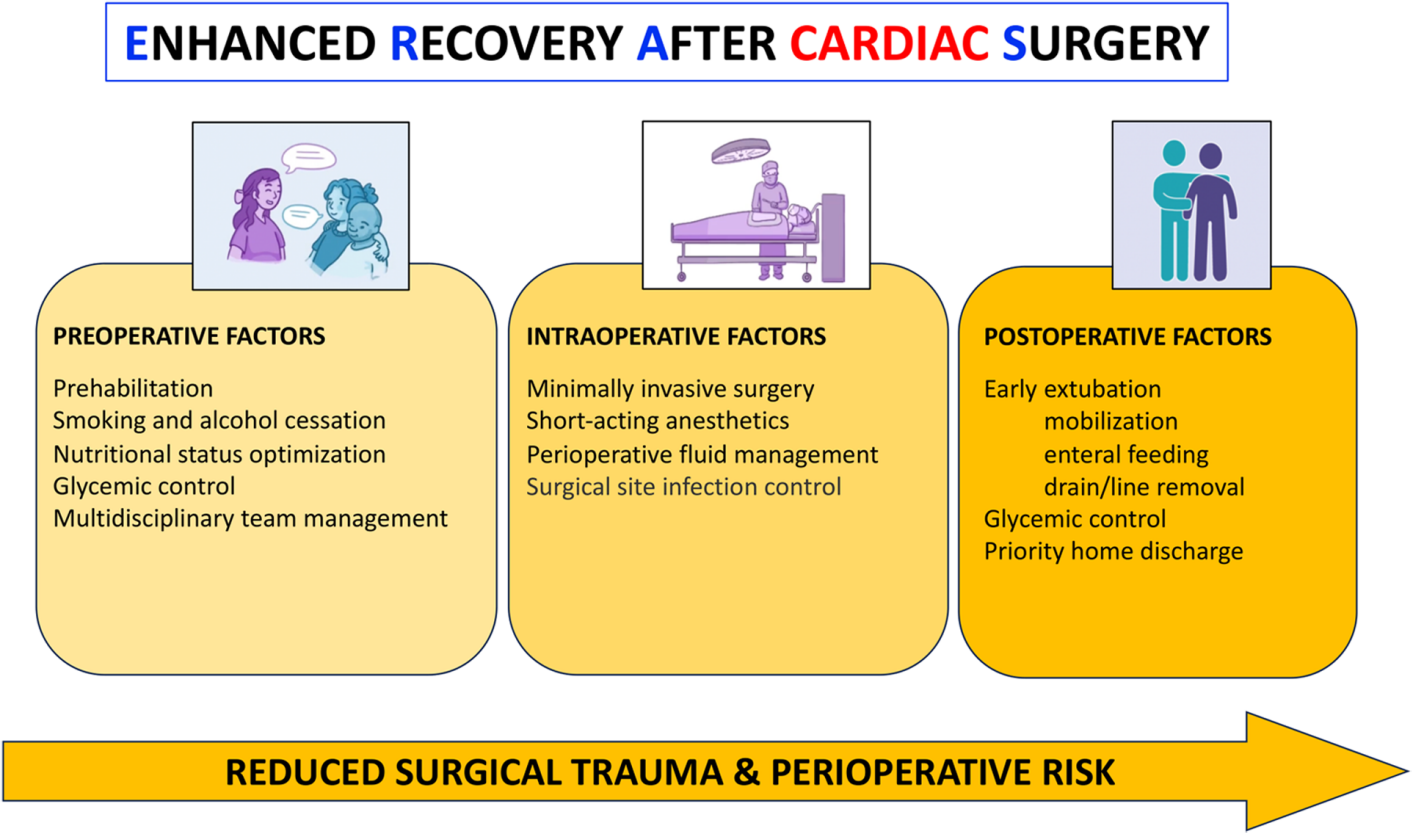
Key points of recent progress in cardiac surgery.

Rather than truly novel techniques, progress in cardiac surgery during the last two decades has greatly relied on patient-specific risk-to-benefit stratification and improved perioperative care (1). Conversely, less invasive surgical approaches and hybrid settings have progressively shifted the traditional definition of “(in)operability”, thereby expanding therapeutic options to higher-risk candidates, most typically elderly individuals with multiple comorbidities (2). The growing interest in this field has promoted the development and implementation of protocols being proposed and aimed at the enhancement of perioperative outcomes. For instance, Enhanced Recovery After Surgery (ERAS) recommends indications for improved pre-, intra-, and postoperative outcomes, across various surgical specialties. More in particular, ERAS also includes pivotal advancements specifically referred to perioperative care in cardiac surgery (Figure 1). Patients undergoing minimally invasive cardiac surgery should represent an ideal cohort. Summarized in the Research Topic devoted to *Peri-operative Care in Cardiac Surgery* are several, albeit rather heterogeneous, efforts and their respective inferences to improve current outcomes in cardiac surgery.

An increasingly relevant topic relates to frail patients. Pozzi et al. reviewed the implications of offering intrinsically higher-risk cardiac operations to this population. They suggested a multidisciplinary approach with the objective of identifying the most vulnerable individuals in order to optimize preoperative conditions, stratify indications according to surgical invasiveness, and promote recovery. Importantly, their report confirms the well-known limitations of 30-day predicted mortality (and morbidity). The latter does not readily translate into a satisfactory medium-to-longer term outcome, and likely underscores overall risk at 3-to-6-months follow-up. Similarly, Gao et al. attempt to implement a home- and hospital-based prehabilitation program, ideally tailored on every single patient, and aimed at optimizing physical performance and alleviating psychological distress before and after cardiac surgery. This potentially promising field requires the contribution and strict collaboration between multiple professional figures, namely, physiotherapists, psychologists, nutritionists, nurses and physicians. The utmost importance of enhanced pain control, including minimally invasive operations, cannot be overemphasized (3).

Renal dysfunction is among the best known and strongest determinants of operative outcome. Zhu et al. better defined the risk of perioperative acute kidney injury (AKI) in relation to longitudinal hemoglobin trajectories and red blood cell transfusion in 4,478 patients from the MIMIC-IV database, outlining the “highest, declining” and “medium, declining” trajectories at reduced risk compared to the “the lowest, rising, and then declining” subgroup. Noteworthily, hemoglobin levels >10 g/dl appear to correlate with a higher risk of AKI irrespective of hemoglobin trajectory, an apparently counterintuitive finding and “hot topic” with respect to liberal vs. restrictive transfusion policies included in more recent guidelines (4). In another retrospective study, Wang et al. also outlined mean platelet volume and cryoprecipitate administration as a risk factor for AKI in adults. Coupled with a significant overall incidence of AKI in recent years, this finding further stresses the importance of a proactive identification of high-risk individuals.

Infection remains a significant complication of the postoperative course following major surgery. The impact on early mortality, prolonged intensive care and hospital stay, and, ultimately, utilization of resources and costs, is particularly relevant in higher-risk patients. Wen et al. analyzed 1,460 patients, regarding the surgical subgroup randomized to coronary artery bypass grafting (CABG) alone (vs. medical therapy or associated ventricular restoration) in the STICH trial, i.e., with ischemic cardiomyopathy and ejection fraction ≤35%. They reported a non-negligible 10.2% incidence, indicating an increased susceptibility to postoperative infection in this scenario. Among other multivariable predictors, they also outlined body mass index, a finding inconsistent with the so-called obesity paradox concept, when extended to morbidity (5). They also identified associated mitral valve procedures as risk factor for postoperative infection. Unlike the well-defined benefits of open surgical repair vs. interventional procedures in degenerative disease with mitral prolapse etiology of regurgitation, the issues regarding when and how to treat ischemic and functional insufficiency remains largely undefined (6–8).

CABG is nowadays less prevalent to address coronary heart disease, primarily in relation to the tremendous achievements of percutaneous technologies and newer-generation stents. Diabetes, however, still portends disappointing results, particularly on the long term, and remains an Achille's heel of interventional cardiology, resulting in CABG being currently offered to patients with extensive and complex coronary anatomy, well reflected by the SYNTAX criteria (9). The identification of outcome predictors, aimed to mitigate the higher risk in CABG candidates with increasingly severe atherosclerotic burden and metabolic disorders, is pivotal. Accordingly, debate continues regarding the optimal choice between on- vs. off-pump, coupled with the increasing role of minimally invasive CABG and hybrid revascularization strategies (10). The report by Salikhanov et al. typically exemplifies this up-to-date scenario. With the routine use of pre- discharge control computed tomography angiography in 439 consecutive patients undergoing isolated on-pump or off-pump CABG, they found that the number of distal anastomoses and the duration of surgery tend to influence the risk of early graft occlusion. This result is not too surprising and likely reflects the technical difficulties and the need for multiple more peripheral grafts on smaller target arteries to achieve complete revascularization in the presence of diffuse coronary atherosclerosis. Knochenhauer et al. outlined in 4,186 patients undergoing isolated CABG that a poor diabetic status, defined as baseline HbA1c >6.5%, anticipated a higher incidence of impaired wound healing, but not deep sternal wound infection The authors concluded that the contraindication for bilateral internal mammary artery on the basis of impaired glycemic control appears unjustified. Rather, further research should be directed to better identify special subgroups of patients at particular risk for deep sternal wound infection. In the conundrum of inflammatory biomarkers, Oh et al. demonstrated a strong relationship between C-reactive protein-to-albumin ratio and one-year mortality following off-pump CABG in 2,082 patients. Not confined to CABG only, Bello et al. characterized perioperative alterations of the acute phase plasma proteome to predict all-cause one-year mortality, hospital length of stay and periprocedural myocardial infarction and stroke in 192 adult patients undergoing on-pump cardiac operations. Among 402 quantified proteins, three were identified as hit-proteins for all endpoints, whereas insulin-like growth factor binding protein 2, IGFBP2, independently showed an over ten-fold association one-year death.

Finally, Li et al. evaluated the incidence and risk factors for gastrointestinal bleeding in a large pediatric population, comprising 21,893 patients who underwent cardiac operations on cardiopulmonary bypass during a 7-years span. This fearsome complication was most commonly encountered at the neonatal age, with an incidence of 23%, in patients with pre- and/or postoperative low cardiac output or hepatic dysfunction receiving complex reconstructive congenital heart surgery, and correlated with longer hospital stay and higher mortality rates. The occurrence of this complication steadily declined to 2% and 0.5% in infants and children, respectively. Baseline multivariable predictors included age and lower weight at time of surgery, likely associated with premature birth. The authors developed a promising prediction model with a sensitivity of 81% and specificity of 84%.

In summary, the Research Topic, which also illustrates some peculiar settings of extracorporeal life support technology (Daughtry and Richardson, Boskovic et al.), an expanding field with a growing role for the intensivist (11, 12), highlights a variegated scenario. More specifically, it touches different areas of current clinical research aimed at the continuing improvement of quality of care in cardiac surgery, largely dependent on optimized perioperative care.
